# Parenting and Future Anxiety: The Impact of Having a Child with Developmental Disabilities

**DOI:** 10.3390/ijerph16040668

**Published:** 2019-02-25

**Authors:** Anna M. Bujnowska, Celestino Rodríguez, Trinidad García, Débora Areces, Nigel V. Marsh

**Affiliations:** 1 Faculty of Pedagogy and Psychology, Maria Curie-Sklodowska University, Narutowicza 12, 20-004 Lublin, Poland; 2 Faculty of Psychology, University of Oviedo, Plaza Feijoo s/n, 33003 Oviedo, Asturias, Spain; rodriguezcelestino@uniovi.es (C.R.); garciatrinidad@uniovi.es (T.G.); arecesdebora@uniovi.es (D.A.); 3 Faculty of Padre Osso, Prado Picon, S/N, 33008 Oviedo, Asturias, Spain; 4 Department of Psychology, James Cook University, 149 Sims Drive, Singapore 387380, Singapore; nigel.marsh@jcu.edu.au

**Keywords:** developmental disabilities, parenting, future anxiety, families

## Abstract

This study examined differences in future anxiety (FA) among mothers and fathers of children with and without developmental disabilities (DD), and it also analyzed differences in FA within the group of parents of children with DD taking into consideration parent-related factors and child-related factors. A group of 167 parents of children with DD were compared to a group of 103 parents of children with typical development. The group with DD included children with autism spectrum disorders, sensory disorders, and intellectual disability. Parents completed the Future Anxiety Scale-FAS1. Mothers of children with DD had a higher general level of FA than fathers of children with and without DD. Mothers of children with DD reported higher anxiety about their future health and the meaning of their future life than fathers of children with DD. For parents of children with DD, those with lower education, male children, and older children reported higher FA. The group at risk of highest general FA are mothers of children with DD, especially those without a professional career. Similarly, parents of teenagers and/or sons with DD are at increased risk of FA.

## 1. Introduction

Two kinds of attitudes may be adopted as a result of thinking about the future: positive or negative. Thus, the feelings tied closest with thinking about the future are hope and anxiety. They are interconnected and their intensity may vary. Domination of one kind over the other results in an individual being either pessimistic or optimistic [1].

The motivational model of hope and fear offered by Zaleski [2,3] concludes that a cognitive representation of future events as positive raises hope, which in turn makes the individual focus on activities leading to the completion of a goal. The negative attitude, on the other hand, results in fear of the future, which may appear long before the event occurs [2]. Fear about the future embodies: cognitive basis for future anxiety and subjective criteria for assessing the level of fear that is caused by the vision of the future and the level of uncertainty [4]. The fear experienced is overt and conscious, and it is caused not by the actual events, but by cognitive representations of the future. Fear is experienced here and now, yet it refers to future events [1]. Moreover, the cognitive representation of future circumstances and events is accompanied by negative emotions [3]. Lack of certainty about the future is related entirely to the individual’s expectations, hopes, and unpredictability of events [1]. For the purposes of this study, future anxiety (FA) is defined as “a negative emotional state and the experience of fears, uncertainty and threats connected with the subjective representation of events and states in a distant future” [4] (p. 172). The many events that can be linked to FA share the aspects of potential loss, and damage or failure that a person considers possible and related to themselves [5].

### 1.1. Future Anxiety in Parents of Children with DD

The presence of FA in parents, especially parents of children with developmental disabilities (DD) has not been studied extensively. However, consideration of studies related to general anxiety of parents, parenting stress and depression may usefully inform consideration of FA.

Parents of children with DD can be considered to be in a unique psychosocial situation. Findings from earlier research indicates that these parents, compared with the parents of children with typical development (TD), report higher levels of stress, risk of depression [6], and stronger feelings of pessimism about the future [7]. Mothers of children with intellectual disabilities (ID) were significantly less happy [8], and parents of children with Autism Spectrum Disorder experienced higher levels of anxiety and stress [9,10] and also more parental stress than parents of children with TD or children with other clinical conditions such as Down syndrome, behavior disorders, ID [11,12]. In general, mothers and fathers raising children with DD report poorer physical health than parents of children with TD [7,13,14]. The limited previous research on FA in parents of children with DD showed that mothers’ anxieties were primarily connected with the child: his or her quality of life in the future, self-reliance, education, relationships with others, possibilities of employment, or financial prospects [15,16,17,18,19,20,21,22,23,24,25].

### 1.2. Factor Related to Future Anxiety in Parents of Children with DD

An individual’s level of FA is a function of three factors: (1) the individual’s susceptibility to anxiety reactions and their tendency to react with fear and anxiety; (2) the individual’s past experiences and the way they assess them; the more difficulties one has encountered, the higher the tendency to react with anxiety; (3) the historical moment and current events. Therefore, people who generally exhibit higher anxiety levels and whose experiences are rather negative, reveal higher intensity of FA especially upon the emergence of new threats [1], such as a new disabled child in the family.

Previous studies indicate that, in the case of parents of children with disabilities, the severity of depressive symptoms, anxiety, worry, pessimism or low quality of life may be related to the characteristics of both the parent and the child. Parent-specific factors which may be related to level of FA in parents include gender, age, education, professional situation, relationship status [6,9,12,26,27,28,29,30,31,32,33]. Child-specific factors which may reduce or intensify FA in parents include the child’s age and type of disability [26,34,35,36,37].

### 1.3. Negative Effect of High Levels of Future Anxiety

A high level of FA may affect negatively the functioning of the parents both on the cognitive and the executive level. On the cognitive level parents may not consider the future as a field for new achievements and have lower expectations as far as future positive events are concerned. On the executive level, FA may result in protective and preventive actions whose aim is to protect what they have in order to maintain the status quo, a reluctance to undertake risky thus creative activities, and an adherence to well-known ways and places, as well as the employment of routine methods to solve life problems [5]. Therefore, perception of the future in parents of children with DD seems to be crucial in determining the parents’ psychosocial functioning and the associated quality of life for the children. Also, hope is a protective factor against psychological distress as it is associated with a variety of positive psychosocial and health outcomes, as well as with lower levels of depression and anxiety [18,38]. The literature suggests that parents of children with disabilities who have a generally optimistic perception of the future are less prone to depression and burnout [38]. Moreover, determining the intensity of FA in parents of children with DD, as well as identifying its dimension, may provide important information for preventive treatment.

### 1.4. Aims of This Study

The purpose of this study was to find answers to the following questions: What are the differences in the intensity of FA in mothers and fathers of children with DD in comparison to mothers and fathers of children with TD? What are the differences in the intensity of FA within the group of parents of children with DD taking into consideration parent-related factors (gender, age, education, marital status, number of children in family, professional activity of parents) and child-related factors (gender, age, type of disability)? The results may provide a starting point to identify groups of parents requiring support, areas of intervention, and to seek methods of reducing FA. In this study, the term developmental disabilities (DD) is used to include intellectual disabilities (ID), cerebral palsy, sensory disorders (SD), genetic disorders such as Down syndrome and other developmental delays [6], and Autism Spectrum Disorders (ASD), which, according to DSM-5, includes both Autistic Disorder and Asperger’s Disorder.

## 2. Materials and Methods

### 2.1. Participants

A total of 328 parents responded to the request to participate in the study but 58 (18%) parents did not complete all questionnaires. The remaining 270 parents constitute the sample reported on here. The parents were recruited through invitation letters distributed by directors of 13 educational institutions in Eastern Poland such as: kindergartens, primary schools and special centers and schools for disabled children. Inclusion criteria for parents were: (a) at least one child aged between 3 and 16 years of age; and (b) the child was living at home. For parents with children with DD the criteria included that their child was diagnosed with ASD, SD, or ID with comorbid disorders such as: visual disorders, cerebral palsy, Down syndrome, and chronical illness (diabetes, epilepsy). The diagnosis of children with DD was confirmed by the psychological and medical documentation existing at their school or kindergarten. The parents were divided into two groups of parents of children with DD (*n* = 167) and parents of children with TD (*n* = 103). 

The difference in the average number of children between the parents of children with DD (M = 1.49, SD = 0.81, range = 1–7 children) and parents of children with TD (M = 1.60, SD = 0.72, range = 1–4 children) was not statistically significant, t(268) = −1.20, *p* > 0.05. A comparison between the two groups on other demographic variables are showed in Table 1. There were no statistically significant (*p* > 0.05) differences between the two groups on gender, age group, or relationship status.

The children with DD had a mean age of 8.29 years (SD = 4.10, range = 2–16 years). The majority (*n* = 137, 82%) were male and the remaining 30 (18%) were female. The types of DD for the children were SD (*n* = 47, 28%), ID with comorbid disorders (*n* = 30, 18%) and ASD (*n* = 90, 54%) (Table 1). Parents of children with TD were more likely (71%) than parents of children with DD (53%) to have completed tertiary (postsecondary) education (χ^2^ (1) = 8.74, *p* = 0.003). Similarly, parents of children with TD were more likely (84%) than parents of children with DD (36%) to live in households where both adults were employed (χ^2^ (1) = 59.11, *p* < 0.001).

### 2.2. Measures

Participants completed a semi-structured questionnaire, which collected demographic information. Parents of children with DD also provided information on the child with the disability. All parents completed the Future Anxiety Scale—FAS1 (Appendix A) [1,3]. 

The FAS was developed for adults as a self-report measure of the tendency to think about their own future with anxiety, uncertainty, and aversion as well as to experience a fear of anticipated failures [1]. Zaleski developed five versions of FAS: FAS1 (a 38-item version in Polish), FAS2 (a 56-item version in English, German, Dutch and French), FAS3 (a 25-item version in Polish and English), FAS4 (a 29-item version in Polish and English), and FAS5 (a short 5-item version named the Dark Future Scale).

The present study employed the FAS1 which was developed based on 150 answers obtained from 95 subjects to the following question: ‘‘What do you fear when thinking about the future?’’ [1] (p. 169). Thirty-eight statements were chosen from these answers and formulated as negative or positive statements and supplied by a seven-point Likert scale ranging from 0 (strongly disagree) to 6 (strongly agree). The FAS1 provides 11 scores for 11 independent subscales: general future anxiety, catastrophe, health and wellbeing, restricted freedom, the meaning of life, politics and economy, achievements, pessimism, social relations, helplessness, and isolation. Each of these 11 subscales is an independent dimension in which future anxiety can raise. The values of each subscale, including the general future anxiety subscale, are computed by summing up from three to four items. There is currently no normative data for the FAS1, but higher scores indicate a higher level of future anxiety. The test-retest reliability for 40 subjects after 35 days was 0.85 [1]. “The Polish version of FAS was correlated with other known anxiety questionnaires in separate studies for assessed validity. The correlation coefficients were: with Cattell’s Overt and Covert Anxiety Scale r = 0.48 (*n* = 88), with Spielberger’s STAI r = 0.61 (*n* = 83), with Taylor’s MAS r = 0.64 (*n* = 102), with Beck’s Hopelessness Scale r = 0.41 (*n* = 60) and with Eysenck’s Neuroticism Scale r = 0.60 (*n* = 135). These results indicate that the FA is related to the anxiety sphere of personality and that besides the common variance with other sorts of anxiety it has its own specificity” [1] (p. 170).

In the present sample high internal consistency reliabilities (Cronbach alpha) were found for the entire scale (α = 0.97, *n* = 270) and for each subscale: general future anxiety levels (α = 0.84), health and wellbeing (α = 0.73), restricted freedom (α = 0.74), the meaning of life (α = 0.78), politics and economy (α = 0.76), achievements (α = 0.77), pessimism (α = 0.80), social relations (α = 0.74), helplessness (α = 0.76), and isolation (α = 0.83); except catastrophe (α = 0.56) for which it was low.

### 2.3. Procedure

The contact with parents of children with DD was established through eight special centers for disabled children in Eastern Poland. These included two special education centers for the blind or low vision, one special education center for the deaf and hard of hearing, two centers for children and young people with ID and children with ASD, one special education center for children with severe mental retardation, and two special preschools with units supporting early childhood development programs for children with different types of disabilities. The parents of children with TD were contacted through randomly chosen preschools, two primary schools and one junior high school. 

The procedure was the same for both types of facilities. The researcher issued an invitation to the directors of the centers that included details concerning the aim of the study, the procedure itself, the way the outcomes would be used, and the information that participation in the study was anonymous and not obligatory. The director presented the aim and objectives to the teachers and class tutors during the pedagogical council meeting, and they later invited the parents and informed them in writing about the aim and date of the study. The study took place at the centers during the parents’ meetings after the first semester of the school year. In a designated room, parents were given sets of questionnaires and were asked to fill them in there. Data collection occurred in groups of up to 25 participants, within a 30-minute time frame. After completing the questionnaires, the parents could talk with the researcher individually. The study was conducted in accordance with The Helsinki Declaration of the World Medical Association [39]. Participation in the study was voluntary, and the anonymity and ethical treatment of the data were guaranteed. Protocol was approved by the Ethics Committee of the Asturias Principality-HUCA (Code: CoPraMo240/18).

### 2.4. Data Analysis

Preliminary examination of the data showed that the assumptions (e.g., skewness and kurtosis) required for the use of parametric statistics. 

Statistical analyses were conducted using ANCOVA with type of DD of the child as covariates to examine the differences between both mothers and fathers of children with DD and mothers and fathers of children with TD. Effect size (η^2^) was interpreted as 0.01 = small, 0.06 = medium, and 0.14 = large [40]. 

For the parents of children with DD further analysis was conducted to examine differences between aspects of FA for specific demographic variables. The 11 aspects of FA were compared across the 6 demographic variables of gender of parent, age group (20–40 vs. 41+ years), relationship status (single vs. married/couple), level of education (secondary vs. tertiary), employment status (one or both parents unemployed vs. both parents employed), and number of children (one child vs. more than one child). To examine for differences across demographic variables, *t*-tests for independent groups were used and effect size (Cohen’s d) was interpreted as 0.2 = small, 0.5 = medium, and 0.8 = large [40]. Subsequently, the 11 aspects of FA were compared across the 3 child-related variables of gender of child, age of child (0–6, 7–12, 13+) and type of DD (ASD, SD, ID with comorbid disorders). To examine for differences across gender of child, t-tests for independent groups was used and effect size (Cohen’s d), across age of child, ANCOVA with type of DD of the child as covariates was used and Effect size (η^2^), across type of DD, ANOVA was used and Effect size (η^2^). Post hoc analysis was conducted using the Tukey Test.

All analyses were conducted using IBM SPSS Statistics 24 for Windows (Predictive Solutions Sp. z o.o., 30-017 Kraków, Poland). Differences were considered significant at level of *p* < 0.05.

## 3. Results

### 3.1. Differences in Future Anxiety between Parents

The differences among the four groups of mothers and fathers of children with and without DD were not statistically significant on 5 of the 11 FAS subscales. The five subscales on which the differences were not significant were catastrophe, restricted freedom, politics and economy, achievements, and isolation (Table 2).

There was a significant effect for group on the general future anxiety, F(3, 266) = 3.54; *p* = 0.008; η^2^ = 0.051 (Table 2). Post hoc tests showed that there was a statistically significant difference between mothers and fathers of children with DD (*p* = 0.035), and between mothers of children with DD and fathers of children with TD (*p* < 0.005). Therefore, on average, mothers of children with DD scored higher on general anxiety about the future than fathers of either group of children.

There was a significant effect for group on the health and wellbeing FA, F(3, 266) = 3.41, *p* = 0.010; η^2^ = 0.049 (Table 2). Post hoc tests showed that there was a statistically significant difference between mothers and fathers of children with DD (*p* = 0.002). Therefore, on average, mothers of children with DD reported higher anxiety about their future health and wellbeing than fathers of children with DD.

There was a significant effect for group on the meaning of life future anxiety, F(3, 266) =3.24; *p* = 0.013; η^2^ = 0.047 (Table 2). Post hoc tests showed that there was a statistically significant difference between mothers and fathers of children with DD (*p* = 0.006). Therefore, on average, mothers of children with DD reported higher anxiety about their future meaning of life than fathers of children with DD. 

There was a significant effect for group on the pessimism FA, F(3, 266) = 2.77; *p* = 0.028; η^2^ = 0.028 (Table 2). Post hoc tests showed that none of the between group comparisons were statistically significant. However, an examination of the means shows that, on average, mothers of children with DD reported higher anxiety about their future pessimism than the other three groups.

There was a significant effect for group on the social relations FA, F(3, 266) = 2.42, *p* = 0.049; η^2^ = 0.026 and the helplessness FA scores, F(3, 266) = 2.81, *p* = 0.026; η^2^ = 0.03 (Table 2). On post hoc tests none of the between group comparisons were statistically significant. However, an examination of the means shows that, on average, mothers of children with DD reported higher anxiety about their future social relations and future helplessness than the other three groups.

### 3.2. Differences among Parents of Children with DD

#### 3.2.1. Parents Variables

For demographic variables only gender of parents of children with DD and their level of education were statistically significant (Table 3). 

Gender differences were significant for 7 of the 11 aspects of future anxiety: general future anxiety (*p* = 0.010), health and wellbeing (*p* < 0.001), the meaning of life (*p* = 0.001), politics and economy (*p* = 0.033), pessimism (*p* = 0.014), social relations (*p* = 0.014), and helplessness (*p* = 0.020). The effect sizes for health and wellbeing, and the meaning of life were medium, while the effect sizes for the other five significant differences were small in magnitude. For all comparisons, women reported higher levels of FA than men (Table 3).

Moreover, level of education differences were significant for 10 of the 11 aspects of future anxiety: general future anxiety (*p* = 0.001), catastrophe (*p* = 0.029), health and wellbeing (*p* < 0.001), the meaning of life (*p* = 0.002), politics and economy (*p* = 0.002), achievements (*p* = 0.035), pessimism (*p* = 0.015), social relations (*p* = 0.008), helplessness (*p* = 0.009), and isolation (*p* = 0.025). The effect sizes for general future anxiety, health and wellbeing, and politics and economy were medium, while the effect sizes for the other seven significant differences were small. The group with lower education reported higher levels of future anxiety (Table 3).

#### 3.2.2. Gender of the Child with DD

The differences for gender of the child with DD were significant for 7 of the 11 aspects of the parents’ future anxiety: general future anxiety (*p* = 0.022), restricted freedom (*p* = 0.012), the meaning of life (*p* = 0.046), achievements (*p* = 0.002), pessimism (*p* = 0.034), helplessness (*p* = 0.039), and isolation (*p* = 0.033). The effect sizes for restricted freedom and achievements were medium, while the effect sizes for the other five significant differences were small. For all comparisons, parents of boys reported higher levels of future anxiety than parents of girls (Table 4).

#### 3.2.3. Age of the Child with DD

The parents were divided into three groups based on the age of the child with the developmental disability: aged 0–6 (*n* = 61), 7–12 (*n* = 74), and 13+ (*n* = 32) years. The differences between parents for the three age groups of children with disabilities were statistically significant on three of the 11 FAS subscales (Table 5).

First, age group showed significant effect on the parents’ general future anxiety scores, F(2, 164) = 3.64; *p* = 0.014; η^2^ = 0.063. Post hoc tests showed that there was a statistically significant difference between the 13+ years age group (M = 14.31, SD = 4.19) and both the 0–6 years (M = 10.57, SD = 6.00; *p* = 0.005) and 7–12 years age group (M = 11.15, SD = 5.26; *p* = 0.016). On average, parents of the older age group reported higher general anxiety about the future than parents of the other two age groups (Table 5).

The second significant effect was on the parents’ health and wellbeing future anxiety scores, F(2, 164) = 2.80, *p* = 0.042. The effect size was small (η^2^ = 0.032). Post hoc tests showed that there was a statistically significant difference between the 13+ years age group (M = 10.97, SD = 3.18) and the 7-12 years age group (M = 8.80, SD = 3.97; *p* = 0.037). The score for the parents of the 0–6 years age group (M = 8.89, SD = 4.73) was not significantly different from either of the other two age groups. On average, parents of the older age group reported higher anxiety about their future health and wellbeing than parents of the middle age group (Table 5).

Finally, the last significant effect was on the parents’ restricted freedom future anxiety scores, F(2, 164) = 3.00, *p* = 0.032. The effect size was small (η^2^ = 0.043). Post hoc tests showed that there was a statistically significant difference between the 13+ years age group (M = 9.75, SD = 3.31) and the 0-6 years age group (M = 7.57, SD = 4.14; *p* = 0.024). The score for the parents of the 7–12 years age group (M = 7.89, SD = 3.60) was not significantly different from either of the other two age groups. On average, parents of the older age group reported higher anxiety about restrictions on their freedom in the future than parents of the youngest age group (Table 5).

#### 3.2.4. Type of DD of the Child

The parents were divided into three groups based on the type of DD of the child: ASD (*n* = 90), SD (*n* = 47), ID with comorbid disorders (*n* = 30). The differences between parents for the three types of groups of children with disabilities were statistically significant on 3 of the 11 FAS subscales (Table 5).

There was a significant effect for disability group on the parents’ catastrophe future anxiety scores, F(2, 164) = 4.74, *p* = 0.010. The effect size was small (η^2^ = 0.055). Post hoc tests showed that there was a statistically significant difference between the SD group (M = 6.53, SD = 3.79) and both the ASD (M = 8.37, SD = 3.68; *p* = 0.019) and ID with comorbid disorders (M = 8.80, SD = 3.79; *p* = 0.027) groups. On average, parents of the SD group reported lower anxiety about future catastrophes than parents of the other two disorder groups (Table 5).

There was a significant effect for disability group on the parents’ restricted freedom future anxiety scores, F(2, 164) = 3.94, *p* = 0.021. The effect size was small (η^2^ = 0.046). Post hoc tests showed that there was a statistically significant difference between the SD group (M = 6.83, SD = 3.97) and the ASD (M = 8.63, SD = 3.57; *p* = 0.023) group. The score for the parents of the ID with comorbid disorders (M = 8.67, SD = 3.93) group was not significantly different from either of the other two disability groups. On average, parents of the SD group reported lower anxiety about future restrictions on their freedom than parents of the ASD group (Table 5).

## 4. Discussion

The principal aim of the present study was to identify the differences in FA intensity between the four groups of mothers and fathers of children with and without DD. A second aim was to examine for differences in FA across demographic variables within the group of parents of children with DD. While previous research has addressed different aspects of the psychosocial functioning of parents of children with DD very little research has focused on FA specifically.

The results confirmed that differences exist between the intensity of general FA with mothers raising a child with DD reporting higher average levels than either fathers raising children with DD or fathers raising children with TD.

Previous research indicates that mothers and fathers, in particular mothers, raising children with different types of disability are characterized by a higher sense of anxiety, face health problems, or have greater pessimism [6,8,9,10,14,29,41,42] compared to parents of children without DD. None of this research has examined future anxiety specifically but has generally reported that both parents report adverse psychosocial consequences of having a child with DD. The current findings show that when parents of children with DD consider the future, mothers report more concerns than fathers, Women bringing up children with DD, in addition to having stronger general anxiety about the future, typically have a greater anxiety about health, wellbeing, and the meaning of life, and also higher pessimism, helplessness, and more serious concerns about social relations. These results would suggest that within families of children with DD there is something specific about the role of the mother that results in greater concerns about the future. Hence, we also examined for the impact of other demographic factors within the group of parents of children with DD.

It was found that the level of FA in parents with a child with DD is affected by the parent-related factors of gender and education level, and the child-related factors of gender, age, type of disability. The strongest influence of a parent’s gender was reported with reference to anxiety about health and wellbeing and meaning of life. Thus, women raising children with DD are much more worried than fathers about their own health, they fear a sudden accident or illness that would cause their inability to take care of the child, they fear being old or being a burden for their families in future. Such kinds of anxieties in women raising children with disabilities are confirmed by studies on the quality of life of disabled children’s parents, indicating that mothers of children with DD displayed lower physical health, impairment in social relationships and in psychological state, and a worse overall perception of quality of life and health [12]. Furthermore, mothers indicated feeling anxious, depressed, and emotionally drained [9,32,33,43].

Another kind of anxiety more frequently faced by mothers of children with DD than their fathers, concerns inability to accomplish their own life goals, which may lead to assessing their own lives as aimless. That less advantageous situation of mothers of children with DD, compared to that of their fathers is confirmed by numerous studies conducted both on parental burnout and stress in parents of children with DD, level of depression and fear, as well as quality of life of parents of children with different kinds of disabilities [12,31].

The current results suggest that the level of education of parents of a child with DD determines the intensity of FA differences between the two levels of education were significant for all the dimensions of future anxiety, excluding restricted freedom. The current results reveal that parents of children with DD with secondary or lower level of education report significantly higher intensity of FA, compared to parents of children with DD with a tertiary level of education. The strongest influence of the level of education for parents of children with DD was reported in the areas of: general anxiety, health and wellbeing, and politics and economy.

Reports by other researchers confirm that with an increase in education level the parental awareness of child rehabilitation requirements, as well as sensitivity to his or her needs may also increase [44]. A higher education level can be treated as an indicator of general knowledge and being acquainted with the issues of raising a disabled child. Parents with higher levels of education and from higher socioeconomic backgrounds are more likely to recognize unusual developmental patterns and seek professional care [45]. In addition, higher educated mothers are less anxious, have more personal resources, better possibilities in the labor market, and support-seeking skills [8]. Maternal education is also related to measures of maternal well-being [8,26], more effective coping with difficulties and burdens resulting from taking care of a disabled child, and therefore, lower intensity of FA.

Gender of the disabled child is an important predictor of the intensity of FA experienced by their parents. Parents who raise a disabled son report significantly higher intensity of future anxiety compared to those bringing up a disabled daughter. This difference was significant for general future anxiety and six of the ten specific areas of future anxiety.

These findings may be the result of two factors. Firstly, intense behavioral disorders and lowered communicational skills occur more frequently in disabled boys than girls and this is associated with higher parental depressiveness, pessimism, and lower quality of life [28,46,47]. Secondly, having a child of a particular sex may evoke specific expectations concerning the social role ascribed to them and the manner of fulfilling it. The social expectations concerning a male are still mainly connected with areas requiring intellectual and/or physical aptitude, which is mainly related to providing for the family [48,49]. Previous studies have reported that a disabled son decreases the father’s self-esteem more than if a disabled daughter [48]; for mothers, that situation also lowers her self-esteem as well as it increases her fear of a negative assessment from other people [49]. The age of the child with DD was found to affect the intensity of parental general future anxiety, their worries about health and wellbeing, and their concerns about restrictions on their freedom in the future. Parents of teenagers with DD, compared to parents of younger children, experience higher FA. These results are similar to those reported by Ogston [18] and Gray [16]. Possibly, the older the child is, the more often the hope for improvement in his/her health is replaced by FA. All possible methods of improving the child’s functioning have already been used up. The parents are now confronted with the actual state of the child with DD, his/her possibilities and restrictions, the consequence of which is fear of the child’s future fate, especially after his/her parents die. 

Type of disability affected the level of anxiety of parents raising children with DD only in two areas of FA. Parents with children with SD (blindness, visually impaired, deaf, and hard of hearing) declared a significantly lower level of anxiety over global disasters than parents raising children with either ASD or ID with comorbid disorders. Parents of children with SD reported less anxiety about restricted freedom than parents of children with ASD. 

Perhaps, the level of anxiety about the future is associated not so much with the kind of disability, as with the level of functioning of the child and the severity of the disorder. According to previous studies, parents of children who were higher functioning had higher hope, lower dispositional worry, and lower future-related worry [18,26]. Therefore, it may be more reasonable in future studies to take into account the criterion related to the level of the child’s functioning and communication abilities than with the type of his/her disability.

## 5. Conclusions

The current study is one of few to examine FA in parents of children with DD. The results suggest that those at greatest risk of high FA are mothers of children with DD and parents without a tertiary level of education raising teenage sons with ASD or with ID. Particularly unfavorable situations can occur when the level of FA exceeds the level of hope. Then, such a state can be detrimental to the parents’ development and functioning. Severe anxiety about the future causes people to have difficulty making vital decisions, and their functioning in everyday life can be subject to considerable disorganization [4]. In addition, anxiety absorbs energy which is needed to tackle problems and limits the possibility of taking action whose results can be creative or therapeutic [5]. Future research into the FA should examine the level of hope and optimism in this group of parents. Obviously, at a low level, anxiety may be beneficial and strengthening. Therefore, it is only reasonable to find out what level of anxiety in parents of children with DD is motivational, and what becomes destructive. Previous family studies have identified the importance of parents and child gender in studies of parental practices towards TD children [50,51,52,53]; based on this, future studies could benefit from including the parental practices of mothers and fathers of child with DD to build a more specific model of psychological intervention.

The applicability of the current findings can be used in three areas of interventions to reduce the level of FA: first, in the personal resources of parents of children with DD, especially mothers; second, in the acceptation of the child’s disability, especially if the child is a son; and third, in families with teenagers or young adults with DD. 

However, some limitations of the study should be considered in future research. First, the sample size must be expanded to check whether the evidence shown by the present results is maintained or not. In addition, in future research lines, it would be desirable to make factorial analysis with more representative sample to clarify the relationship between all variables analyzed, as well as to analyze the possible interactions between the demographic variables. Also, the statistical design used in our analysis may cause the risk of a type II error. It may be useful to get information on the child’s level of functioning in additional to type of DD.

## Figures and Tables

**Table 1:** Comparison of parent demographic variables for the two groups of children with developmental disabilities (*n* = 167) and children with typical development (*n* = 103).

Variable	Group		χ^2^
	Disability	No Disability	
Gender			
Female	108 (65%)	73 (71%)	1.11
Male	59 (35%)	30 (29%)	
Age			
20-40 years	120 (72%)	70 (68%)	0.46
41+ years	47 (28%)	33 (32%)	
Relationship status			
Single	22 (13%)	12 (12%)	0.13
Married/couple	145 (87%)	91 (88%)	
Level of education			
Secondary	79 (47%)	30 (29%)	8.75 *
Tertiary	88 (53%)	73 (71%)	
Employment			
One or both unemployed	106 (64%)	16 (16%)	59.11 *
Both employed	61 (36%)	87 (84%)	

* *p* < 0.05.

**Table 2:** Means, standard deviations, F-statistics, and effect sizes for ANCOVAs of parents of children with developmental disabilities and parents of typically developing children.

Future Anxiety	Disability						No Disability							
	Mothers			Fathers			Mothers			Fathers				
	(*n* = 108)			(*n* = 59)			(*n* = 73)			(*n* = 30)				
	M	SD		M	SD		M	SD		M	SD	F	η^2^	Post hoc
General future anxiety	12.35	5.54		10.07	5.17		11.00	5.02		8.87	4.20	3.54 *	0.051	1 > 2,4
Catastrophe	8.32	4.00		7.20	3.34		8.30	5.60		7.37	2.88	0.98	--	--
Health and wellbeing	10.07	4.03		7.73	4.11		9.37	4.06		9.30	3.58	3.41 *	0.049	1 > 2
Restricted freedom	8.54	3.82		7.39	3.72		7.64	3.63		6.97	3.76	1.57	--	--
The meaning of life	8.32	4.01		6.27	3.38		7.27	3.94		6.57	3.65	3.24*	0.047	1 > 2
Politics and economy	9.66	3.99		8.24	4.27		9.00	3.85		8.83	2.38	1.40	--	--
Achievements	12.22	4.23		10.83	4.90		11.18	5.17		11.53	3.45	1.12	--	--
Pessimism	9.18	4.03		7.58	3.84		7.89	3.81		7.27	3.02	2.77 *	0.028	--
Social relations	12.94	4.64		11.08	4.59		12.55	5.02		10.77	2.98	2.40*	0.049	--
Helplessness	8.69	3.72		7.27	3.76		8.45	3.64		6.73	3.50	2.81 *	0.026	--
Isolation	10.41	5.24		9.17	4.93		9.19	4.90		8.27	4.70	1.74	--	--

Note. Post hoc = 1 (mothers of children with DD); 2 (fathers of children with DD); 4 (fathers of TD children). * *p* < 0.05.

**Table 3:** Means, standard deviations, *t*-statistics (df = 165), and effect size (Cohen’s d) for demographic variables and future anxiety for the families with a child with DD.

**Future Anxiety**	**Gender of Parents**						
	**Female (*n* = 108)**			**Male (*n* = 59)**			
	**M**	**SD**		**M**	**SD**	**t**	**d**
General future anxiety	12.35	5.54		10.07	5.16	2.61 *	0.43
Catastrophe	8.32	4.00		7.20	3.34	1.83	--
Health and wellbeing	10.07	4.02		7.73	4.11	3.57 *	0.57
Restricted freedom	8.54	3.82		7.39	3.72	1.87	--
The meaning of life	8.32	4.01		6.27	3.38	3.34 *	0.55
Politics and economy	9.66	3.99		8.24	4.27	2.14 *	0.34
Achievements	12.22	4.23		10.83	4.90	1.92	--
Pessimism	9.18	4.02		7.58	3.83	2.49 *	0.41
Social relations	12.94	4.64		11.08	4.59	2.49 *	0.40
Helplessness	8.69	3.72		7.27	3.76	2.35 *	0.38
Isolation	10.41	5.24		9.17	4.93	1.49	--
**Future Anxiety**	**Parents Education Level**						
	**Secondary (*n* = 79)**			**Tertiary (*n* = 88)**			
	**M**	**SD**		**M**	**SD**	**t**	**d**
General future anxiety	13.06	5.75		10.18	4.92	3.49 *	0.54
Catastrophe	8.61	4.15		7.32	3.38	2.21 *	0.34
Health and wellbeing	10.71	4.15		7.93	3.80	4.51 *	0.70
Restricted freedom	8.44	4.07		7.85	3.57	1.00	--
The meaning of life	8.59	4.07		6.70	3.56	3.20 *	0.50
Politics and economy	10.22	4.17		8.20	3.88	3.23 *	0.50
Achievements	12.51	4.92		11.03	4.01	2.13 *	0.33
Pessimism	9.41	4.23		7.90	3.71	2.45 *	0.38
Social relations	13.29	4.86		11.39	4.36	2.67 *	0.41
Helplessness	9.00	4.12		7.47	3.31	2.63 *	0.41
Isolation	10.92	5.71		9.11	4.44	2.27 *	0.36

* *p* < 0.05.

**Table 4:** Means, standard deviations, *t*-statistics (df = 165), and effect size (Cohen’s d) for gender of child and future anxiety of parents with children with DD.

	Gender of Child						
Future Anxiety	Female (*n* = 27)			Male (*n* = 140)			
	M	SD		M	SD	t	d
General future anxiety	9.33	5.32		11.97	5.45	2.31*	0.49
Catastrophe	7.15	3.71		8.08	3.82	1.16	--
Health and wellbeing	7.96	4.45		9.49	4.12	1.75	--
Restricted freedom	6.44	3.32		8.46	3.83	2.55*	0.56
The meaning of life	6.22	3.63		7.86	3.92	2.01*	0.43
Politics and economy	7.74	4.70		9.43	3.98	1.96	--
Achievements	9.30	4.26		12.20	4.42	3.14*	0.67
Pessimism	7.11	4.15		8.90	3.95	2.14*	0.44
Social relations	11.26	5.03		12.49	4.62	1.25	--
Helplessness	6.81	3.51		8.46	3.78	2.09*	0.45
Isolation	8.04	4.70		10.34	5.16	2.15*	0.47

* *p* < 0.05.

**Table 5:** Means, standard deviations, F-statistics, and effect sizes for ANCOVA of age of the children with DD and ANOVA of type of DD of the child.

**Future Anxiety**	**Age of the Children with DD**								
	**0–6**		**7–12**		**13+**				
	**(*n* = 61)**		**(*n* = 74)**		**(*n* = 32)**				
	**M**	**SD**	**M**	**SD**	**M**	**SD**	**F**	η^2^	**Post hoc**
General future anxiety	10.57	6.00	11.15	5.26	14.31	4.19	3.64 *	0.063	0–6 < 13+ 7–12 < 13+
Catastrophe	7.33	3.91	7.88	3.77	9.19	3.52	1.79	--	--
Health and wellbeing	8.88	4.73	8.80	3.97	10.97	3.18	2.80 *	0.032	7–12 < 13+
Restricted freedom	7.57	4.14	7.89	3.60	9.75	3.31	3.00 *	0.043	0–6 < 13+
The meaning of life	7.05	4.31	7.42	3.59	9.06	3.58	2.02	--	--
Politics and economy	9.03	4.87	8.93	3.49	9.91	4.01	0.51	--	--
Achievements	11.47	5.03	11.57	4.33	12.59	3.88	0.54	--	--
Pessimism	8.33	4.46	8.53	3.70	9.34	3.89	0.51	--	--
Social relations	12.36	5.22	11.89	4.40	13.06	4.32	0.48	--	--
Helplessness	7.77	4.21	8.26	3.43	8.84	3.72	0.67	--	--
Isolation	9.29	5.74	10.08	4.88	11.00	4.48	0.79	--	--
	**Type of DD of the Child**								
	**ASD**		**SD**		**ID with Comorbid**				
	**(*n* = 90)**		**(*n* = 47)**		**(*n* = 30)**				
	**M**	**SD**	**M**	**SD**	**M**	**SD**	**F**	η^2^	**Post hoc**
General future anxiety	11.87	5.36	10.13	5.03	12.80	6.32	2.54	--	--
Catastrophe	8.37	3.68	6.53	3.79	8.80	3.79	4.74 *	0.055	ASD, ID > SD
Health and wellbeing	9.04	3.95	9.02	3.85	10.20	5.31	0.95	--	--
Restricted freedom	8.63	3.57	6.83	3.97	8.67	3.93	3.94 *	0.046	ASD > SD
The meaning of life	7.58	3.83	7.17	3.62	8.33	4.59	0.81	--	--
Politics and economy	9.21	3.70	9.13	4.51	9.03	4.86	0.02	--	--
Achievements	12.10	4.22	10.53	4.69	12.50	4.86	2.43	--	--
Pessimism	8.86	3.80	7.87	4.08	9.03	4.55	1.12	--	--
Social relations	12.31	4.39	11.74	4.65	13.07	5.61	0.73	--	--
Helplessness	8.53	3.58	7.28	4.13	8.60	3.69	1.94	--	--
Isolation	10.26	4.99	9.00	5.07	10.63	5.69	1.22	--	--

* *p* < 0.05.

## Appendix A

**Future Anxiety Scale (FAS1)** **Author: Z. Zaleski**

The statements below concern your attitude toward the future. Read them carefully. If a given statement accurately describes your attitude, indicate number “6” on the attached scale. If the statement is not a true description of your attitude, indicate “0”. Each statement may reflect your attitude to a different degree. Indicate the number that most accurately defines your point of view. There are no “right” or “wrong” answers. All answers are valuable, provided they are sincere. The survey is anonymous and strictly for the purpose of academic research. The scale:

0—Decidedly false; 1—False; 2—Somewhat false; 3—Hard to say; 4—Somewhat true; 5—True; 6—Decidedly true

1. I am worried when I think about my future	0 1 2 3 4 5 6
2. I am afraid that some disaster will happen soon	0 1 2 3 4 5 6
3. I am anxious about the possibility of some accident or a serious illness (e.g. cancer, AIDS)	0 1 2 3 4 5 6
4. I worry that in my life I will be unable to manage my development freely	0 1 2 3 4 5 6
5. I am afraid of the moment when I will have to sum my life up	0 1 2 3 4 5 6
6. I am afraid that change in economic and political situation will be a threat to my future	0 1 2 3 4 5 6
7. I am afraid that I will not be able to overcome problems and troubles piling up	0 1 2 3 4 5 6
8. I am afraid that difficulties related to me will last for a long time	0 1 2 3 4 5 6
9. I am afraid that in future “man to man will be a wolf”	0 1 2 3 4 5 6
10. I am afraid that one day I will not have any influence upon others	0 1 2 3 4 5 6
11. I am afraid that in future I will be negatively assessed by people	0 1 2 3 4 5 6
12. I am trembling with fear at the thought what the next day, month, year will bring	0 1 2 3 4 5 6
13. I feel fear at the thought that life is passing quickly	0 1 2 3 4 5 6
14. I am anxious at the thought that I will not be able to accomplish my goal in future	0 1 2 3 4 5 6
15. Politicians will make wrong decisions bringing irreversible results	0 1 2 3 4 5 6
16. I am worried about the failures I might encounter.	0 1 2 3 4 5 6
17. Even if something goes well in my life, later the fortune will turn its back at me later.	0 1 2 3 4 5 6
18. I am afraid I will not provide my family with good financial conditions	0 1 2 3 4 5 6
19. When I think about the future, I am afraid I will depend on others	0 1 2 3 4 5 6
20. When I think about my future, I am afraid I will not have any friends	0 1 2 3 4 5 6
21. I feel tense and anxious when I think about my future dealings	0 1 2 3 4 5 6
22. I am worried that I will be a burden for someone when I am old	0 1 2 3 4 5 6
23. I am afraid that within a few years I will assess my life as pointless	0 1 2 3 4 5 6
24. When I think about my future, I feel fear of poverty	0 1 2 3 4 5 6
25. I am frightened to face crises and difficulties in life	0 1 2 3 4 5 6
26. I fear that my life will change for worse in future	0 1 2 3 4 5 6
27. I am afraid that in future our society will disintegrate	0 1 2 3 4 5 6
28. I am worried that as time goes by, I will not be able to oppose others	0 1 2 3 4 5 6
29. I am afraid that one day I will be left alone	0 1 2 3 4 5 6
30. I am afraid of the future	0 1 2 3 4 5 6
31. I feel that the world is going to decline	0 1 2 3 4 5 6
32. I am afraid that I will not be appreciated in my profession	0 1 2 3 4 5 6
33. I am afraid that something bad will happen to people who are important to me	0 1 2 3 4 5 6
34. I am afraid that one day I will not be able to make my own decisions any more	0 1 2 3 4 5 6
35. I am worried that in future accidents will happen	0 1 2 3 4 5 6
36. I am afraid that in future I will not be able to meet the requirements I will face	0 1 2 3 4 5 6
37. I am afraid that in future I will not have stable relationships with the others	0 1 2 3 4 5 6
38. I am afraid I will not be able to fulfil myself in future	0 1 2 3 4 5 6

Please check to see if you have answered all the questions. Thank you for your co-operation.
